# High AMH Levels Are Associated With Gestational Hypertension in Patients With PCOS who Underwent IVF/ICSI-ET

**DOI:** 10.1210/clinem/dgae324

**Published:** 2024-05-13

**Authors:** Menghui Zhang, Shujun Liu, Fuli Zhang, Hao Shi, Fang Wang, Caihong Chen, Qingling Yang, Shanjun Dai, Yuling Liang, Yihong Guo

**Affiliations:** Reproductive Medicine Center, First Affiliated Hospital of Zhengzhou University, Zhengzhou, 450052, China; Henan Key Laboratory of Reproduction and Genetics, First Affiliated Hospital of Zhengzhou University, Zhengzhou, 450052, China; Reproductive Medicine Center, First Affiliated Hospital of Zhengzhou University, Zhengzhou, 450052, China; Henan Key Laboratory of Reproduction and Genetics, First Affiliated Hospital of Zhengzhou University, Zhengzhou, 450052, China; Reproductive Medicine Center, First Affiliated Hospital of Zhengzhou University, Zhengzhou, 450052, China; Henan Key Laboratory of Reproduction and Genetics, First Affiliated Hospital of Zhengzhou University, Zhengzhou, 450052, China; Reproductive Medicine Center, First Affiliated Hospital of Zhengzhou University, Zhengzhou, 450052, China; Henan Key Laboratory of Reproduction and Genetics, First Affiliated Hospital of Zhengzhou University, Zhengzhou, 450052, China; Reproductive Medicine Center, First Affiliated Hospital of Zhengzhou University, Zhengzhou, 450052, China; Henan Key Laboratory of Reproduction and Genetics, First Affiliated Hospital of Zhengzhou University, Zhengzhou, 450052, China; Reproductive Medicine Center, First Affiliated Hospital of Zhengzhou University, Zhengzhou, 450052, China; Henan Key Laboratory of Reproduction and Genetics, First Affiliated Hospital of Zhengzhou University, Zhengzhou, 450052, China; Reproductive Medicine Center, First Affiliated Hospital of Zhengzhou University, Zhengzhou, 450052, China; Henan Key Laboratory of Reproduction and Genetics, First Affiliated Hospital of Zhengzhou University, Zhengzhou, 450052, China; Reproductive Medicine Center, First Affiliated Hospital of Zhengzhou University, Zhengzhou, 450052, China; Henan Key Laboratory of Reproduction and Genetics, First Affiliated Hospital of Zhengzhou University, Zhengzhou, 450052, China; Reproductive Medicine Center, First Affiliated Hospital of Zhengzhou University, Zhengzhou, 450052, China; Henan Key Laboratory of Reproduction and Genetics, First Affiliated Hospital of Zhengzhou University, Zhengzhou, 450052, China; Reproductive Medicine Center, First Affiliated Hospital of Zhengzhou University, Zhengzhou, 450052, China; Henan Key Laboratory of Reproduction and Genetics, First Affiliated Hospital of Zhengzhou University, Zhengzhou, 450052, China

**Keywords:** anti-Müllerian hormone, gestational hypertension, *in vitro* fertilization, polycystic ovary syndrome

## Abstract

**Background:**

Patients with polycystic ovary syndrome (PCOS) have a higher risk of obstetric complications. The association between anti-Müllerian hormone (AMH) and gestational hypertension in these patients is poorly understood.

**Objective:**

To determine the association between serum AMH levels and gestational hypertension in patients with PCOS undergoing fresh embryo transfer.

**Methods:**

This retrospective study included 649 patients with PCOS who had singleton live births after undergoing fresh embryo transfers. The association of AMH with gestational hypertension in these patients was estimated before and after propensity score matching.

**Results:**

Patients with gestational hypertension had higher AMH levels than those without gestational hypertension. In single-factor logistic regression, the odds of gestational hypertension increased by 11.7% and 18.6% for every 1ng/mL increase in AMH before and after adjusting for confounding factors (odds ratio [OR], 1.117; 95% CI, 1.025-1.217; *P* = .012; adjusted OR, 1.186; 95% CI, 1.061-1.327; adjusted *P* = .003), respectively. The odds of gestational hypertension increased more than 100% (adjusted OR, 2.635; 95% CI, 1.132-6.137; adjusted *P* = .025) in the 75th percentile group (>9.30 ng/mL) and more than 3 times (adjusted OR, 4.75; 95% CI, 1.672-13.495; adjusted *P* = .003) in the 90th percentile group (>12.31 ng/mL) compared to the without gestational hypertension group. AMH level was still associated with gestational hypertension after propensity score matching. The area under the curve of AMH predicting gestational hypertension was 0.654 (95% CI, 0.532-0.776; *P* = .011) with an optimal cutoff value of 11.975 ng/mL.

**Conclusion:**

High serum AMH level prepregnancy (especially at levels >9.30 ng/mL) indicates a high odds of gestational hypertension in patients with PCOS undergoing fresh embryo transfer.

Polycystic ovary syndrome (PCOS) is one of the most common reproductive diseases affecting women worldwide. PCOS is a heterogeneous condition characterized by polycystic ovarian morphology, ovulation disorders, hyperandrogenism, glucose metabolism disorders, cardiovascular diseases, and obesity (1). Women with PCOS tend to have low fertility and seek assisted reproductive technology (ART). Although ART can improve reproductive outcomes, patients with PCOS have an increased risk of ovarian hyperstimulation during ART and a higher incidence of pregnancy complications (2). Compared with healthy women, women with PCOS have a higher risk of obstetric complications, such as preterm birth, gestational diabetes mellitus, gestational hypertension, multiple pregnancies, and small for gestational age infants (3).

Recent studies have suggested that the relationship between anti-Müllerian hormone (AMH) and female reproductive outcomes should not be ignored. AMH levels in the follicular fluid of a single oocyte can effectively predict the live birth outcome of an intracytoplasmic sperm injection (ICSI) single-embryo transplantation (4). Low AMH may be a risk factor for miscarriage during the in vitro fertilization (IVF) embryo transfer cycle (5). A large retrospective cohort study showed no association between serum AMH and the risk of preterm birth in patients with IVF embryo transfer cycles. When analyzed in the PCOS population, high serum AMH was strongly associated with the risk of preterm birth (6). Although serum AMH levels gradually decrease during pregnancy because of ovarian function suppression (7), the AMH levels of patients with PCOS during pregnancy are higher than that of normal women (8, 9). High doses of AMH injected into mice during late pregnancy showed a high abortion rate and a small litter size (10), indicating that elevated AMH levels during pregnancy may harm fetal growth and development.

Hypertensive disorders of pregnancy (HDPs) are among the leading causes of maternal and perinatal mortality worldwide, accounting for 9% to 26% of the total maternal deaths in different regions (11). HDPs seriously endanger the life and health of pregnant women and newborns and increase the medical and economic burden on society and families. The common clinical problem with HDPs is that they are not recognized early enough to prevent deterioration and organ suffering. Gestational hypertension may be one of the first signs of preeclampsia (PE). In recent years, ovarian reserve has been found to be associated with cardiovascular disease (12, 13). As a female-specific cardiovascular disease, preeclampsia has a positive association with decreased ovarian reserve function (14, 15). Meanwhile, several clinical studies have shown that AMH is associated with cardiovascular and metabolic indicators such as cholesterol, low-density lipoprotein (LDL), and high-density lipoprotein (HDL) (16-18). AMH levels in serum and umbilical cord blood were higher in women with preeclampsia than those without (19, 20). However, there are no studies to focus on the association between prepregnancy basal serum AMH level and hypertension during pregnancy.

Therefore, this retrospective study aimed to interpret the relationship between AMH level and gestational hypertension in patients with PCOS undergoing fresh embryo transfer and evaluate their odds of gestational hypertension to identify the high-risk population.

## Methods

### Participants

This retrospective study included 15 612 patients undergoing controlled ovarian stimulation IVF/ICSI fresh embryo transfer (ET) cycles between January 2016 and December 2020. The follow-up duration was >12 months. Patients with ovarian tumors, a history of ovarian surgery, ovarian endometriomas, uterine anomalies, preexisting diabetes, or hypertension were excluded. Additionally, patients without data regarding the assisted reproductive process, pregnancies, or AMH values were excluded. Patients have a physical examination, including measurement of blood pressure and a detailed medical history, to identify the presence of hypertension when they were first seen in our reproductive center. Patients with hypertension or preexisting hypertension were subsequently excluded. HDPs are the most important maternal complication associated with multiple pregnancies and increase maternal morbidity, fetal and neonatal morbidity, and mortality (21). To exclude the effect of multiple pregnancies/births on the study, multiple gestations were excluded. PCOS was diagnosed according to the modified Rotterdam criteria, which included menstrual abnormalities (menstrual cycle >35 days) combined with either hyperandrogenism (HA) or polycystic ovarian morphology (PCOM) (22). Based on clinical features, PCOS includes 4 subtypes, namely, A, B, C, and D subtypes, which present with HA + PCOM + menstrual abnormalities, HA + menstrual abnormalities, HA + PCOM, and PCOM + menstrual abnormalities, respectively.

Among these, 2126 patients had all embryos frozen because of ovarian hyperresponsiveness, 1341 cancelled their cycles because of other causes, and 12 145 women underwent fresh embryo transfers. Of these women, 1669 had twin and triple pregnancies, 6004 undergoing fresh embryo transfers did not achieve live births, and 4472 had singleton live births. A total of 6004 patients who failed to achieve live births included serum human chorionic gonadotropin (HCG) (−), biochemical pregnancy that did not achieve clinical pregnancy, ectopic pregnancy, and abortion before 28 weeks. In addition, 117 patients with missing pregnancy data were excluded, and 3706 were ineligible for PCOS diagnosis. Accordingly, 649 patients with PCOS and singleton births were analyzed in this study (Supplementary Fig. S1) (23).

There were some missing data in this study, including 3 (0.49%) triglyceride (TG), 2 (0.33%) total cholesterol (TC), 2 (0.33%) HDL, three (0.49%) LDL, 2 (0.33%) diastolic blood pressure (DBP), and 23 (3.75%) fasting blood glucose (FBG), which are all from group without gestational hypertension. And there was only 1 (2.86%) missing data from FBG in the group with gestational hypertension. We used the Little's MCAR test and the result of the test was not significant (χ^2^ = 21.887, *u* = 22, *P* = .467), which indicates that the missing values were missing completely at random (24). We used single mean imputation for missing data in the study (25).

Live birth was defined as delivery after at least 24 weeks of gestation. Pregnancies were classified as preterm (<37 weeks), term (37-42 weeks), or overdue (≥42 weeks) according to gestational age at delivery. Each patient was diagnosed with gestational hypertension by an obstetrician during perinatal care. After 20 weeks of gestation, high blood pressure was first observed with systolic blood pressure (SBP) ≥140 mm Hg or DBP ≥90 mm Hg, measured on 2 occasions at least 4 hours apart at least twice on the same arm without urinary protein. All data were acquired from the electronic medical records of the Reproductive Medicine Center of the First Affiliated Hospital of Zhengzhou University. This study was approved by the institutional review board and ethics committee of the First Affiliated Hospital of Zhengzhou University (approval number 2017-KY-15). Participants provided informed consent to be included in the study.

### Controlled Ovarian Stimulation Protocols

#### Follicular phase GnRH—a long-acting protocol

During days 2 and 3 of the menstrual cycle, patients received intramuscular injections of triptorelin long-acting formulation (dabigatran 3.75 mg; Ferring, Germany). The pituitary gland reached down-regulation at 28 to 42 days (FSH < 5 mIU/mL, LH < 5 mIU/mL, estradiol < 30 pg/mL; *P* < .6 ng/mL; follicle diameter 4-7 mm; endometrial thickness <5 mm). FSH was used to initiate ovarian hyperstimulation (Vonafine, Serono; Purican, The Netherlands; u-FSH, Livzon). The initial dose depended on the clinical characteristics of the patient and the number of antral follicles, whereas subsequent doses were adjusted according to follicular development and hormone levels.

#### Luteal phase GnRH—a short-acting protocol

Patients in the luteal phase received intramuscular triptorelin (Ferring, 0.1 mg, Switzerland; Iposen, 0.1 mg, France). After 10 days, the dose was reduced to 0.05 mg/day. When the pituitary gland reached down-regulation (FSH < 5 mIU/mL, LH < 5 mIU/mL, estradiol < 30 pg/mL; *P* < .6 ng/mL; follicle diameter 4-7 mm; endometrial thickness <5 mm), ovarian hyperstimulation was initiated with FSH.

#### Antagonist protocol

Ovarian stimulation with FSH was started on the second day of the menstrual cycle, and GnRH-A (Szechke, Baxter, 0.25 mg/d) was added between days 6 and 8 of ovulation stimulation until the day of HCG.

#### Stimulation protocol

Ovarian stimulation with oral letrozole was started between days 3 and 5 of the menstrual cycle, and human menopausal gonadotropin was used appropriately according to the growth rate of follicles until the day of HCG.

The methodology for oocyte retrieval, insemination, embryo transfer, and luteal phase support are described in our previous report (26, 27).

### Biochemical Assays

All serum measurements were performed at the Endocrine Laboratory of Reproductive Medicine Center. Blood samples were drawn for analysis for AMH and testosterone before ovarian stimulation either when the endometrial thickness and the diameter of ovarian follicles were both <5 mm and there was no dominant follicle growth, or during the second to fourth days of menstruation. Samples were stored at −20°C until assayed. Hormone levels were measured using a fully automated Elecsys immunoassay analyser (Roche, Germany; AMH: RRID: AB_2895131; testosterone: RRID:AB_2783736; estradiol: RRID:AB_2905575). The intra- and inter-assay coefficients of variation were both <5% for all assays (AMH, T, and estradiol). The assay sensitivity of AMH is 0.049 pmol/L (0.007 ng/mL), which is the 95th percentile of values obtained from 60 measurements derived from several independent measurements of several analytics-free samples.

The FBG, TG, TC, HDL, and LDL were measured by a fully automated biochemical analyzer (Roche, Germany). Fasting insulin was measured by a fully automated immunoassay analyzer ARCHITECT i2000SR (Abbott, USA; RRID: AB_3075437).

### Blood Pressure Measurement

Both SBP and DBP were measured by well-trained nurses using a wrist electronic blood pressure monitor (OMRON HEM-6200) when patients were first seen in our reproductive center. The OMRON monitor was certified by the Association for the Advancement of Medical Instrumentation or the British Hypertension Society. And the accuracy of the wrist blood monitor tested by static detector was within ±3 mm Hg (±0.4 Kpa) (28). If the blood pressure was abnormal, we advised patients to schedule a professional department consultation.

Each patient with gestational hypertension was diagnosed by a professional obstetrician using a standard mercury sphygmomanometer during perinatal care after 20 weeks of gestation. After 20 weeks of gestation, high blood pressure was first observed with SBP ≥140 mm Hg or DBP ≥90 mm Hg measured on 2 occasions at least 4 hours apart at least twice on the same arm.

### Statistical Analysis

Continuous variables are presented as mean values and corresponding standard deviations, and the Student *t*-test was used for comparison. Categorical variables were expressed as frequencies and percentages and compared using the chi-square test. Multivariable logistic regression analysis was used to evaluate the relationship between the serum AMH level and gestational hypertension. Collinearity test was performed before multivariable logistic regression analysis. As shown in Supplementary Table S1 (29), the type and number of embryos transferred, LDL, and TC had serious collinearity problems. We disregarded the number of embryos transferred and TC in the multivariate model. Finally, the multivariate analysis was adjusted for age, body mass index (BMI), testosterone, PCOS phenotype, controlled ovarian stimulation protocols, the type of embryos transferred, mode of insemination, the endometrial thickness at the day of embryo transfer, the serum estradiol levels on the day of HCG injection, total gonadotropin (Gn) dosage, and total oocytes retrieved, and other prepregnancy values including SBP, DBP, TG, HDL, LDL, and FBG. Because the PCOS phenotype is a nominal (nonordinal) categorical variable, we used dummy variables, with type D as the control. Subgroup analysis was conducted by fasting insulin using logistic regression models, and the interaction between subgroups was tested. Receiver operating characteristic (ROC) analysis was used to determine the ability to predict gestational hypertension. The optimal cutoff value of AMH for predicting gestational hypertension was determined based on the Youden index.

Propensity score matching (PSM) was used to identify women with gestational hypertension similar to those with normal blood pressure. This was performed using a logistic regression model. Age, BMI, the type of embryos transferred, mode of insemination, SBP, DBP, PCOS phenotype, and FBG prepregnancy values were included in the PSM model. The caliper value was 0.2. Patients with gestational hypertension were matched to those without gestational hypertension at a 1:4 ratio with the nearest neighbor matching algorithm by random matching order.

Statistical analyses were performed using IBM SPSS Statistics for Windows (version 26.0. Armonk, NY: IBM Corp.). All tests were 2-sided, and a *P* value < .05 was considered statistically significant. GraphPad Prism for Windows (version 8.3.0, GraphPad Software, San Diego, CA, USA; www.graphpad.com) was used to generate the ROC curves.

## Results

### Clinical Characteristics of Patients With PCOS

The baseline data of patients with and without gestational hypertension were retrospectively analyzed. In these fresh IVF/ICSI cycles, compared to patients without gestational hypertension, patients with gestational hypertension had a higher rate of cesarean delivery, younger gestational age, higher BMI, higher serum AMH, and lower serum estradiol levels on the day of HCG injection than patients who did not have gestational hypertension (all *P* < .05). The patients with gestational hypertension also had higher SBP, DBP, and FBG (*P* < .05). The gestational hypertension group had much more PCOS phenotype A, but the difference had no statistical significance (*P* = .069). Age, testosterone, controlled ovarian stimulation protocols, the type and number of embryos transferred, mode of insemination, the endometrial thickness on the day of embryo transfer, total Gn dosage, total oocytes retrieved, TG, TC, HDL, and LDL were comparable between the 2 groups (*P* > .05) (Table 1).

**Table 1.** Demographic characteristics of PCOS patients with gestational hypertension and without gestational hypertension before and after propensity score matching

	Before PSM			After PSM		
Variables	With gestational hypertension (n = 35)	Without gestational hypertension (n = 614)	*P^a^*	With gestational hypertension (n = 29)	Without gestational hypertension (n = 111)	*P^a^*
Age (y)	29.57 ± 3.5	28.49 ± 3.5	.078	29.31 ± 3.40	29.16 ± 3.56	.841
BMI (kg/m^2^)	26.19 ± 3.1	24.05 ± 3.4	.000	25.70 ± 2.77	25.45 ± 3.61	.697
Testosterone (ng/mL)	0.47 ± 0.2	0.67 ± 4.2	.781	0.47 ± 0.24	0.99 ± 5.90	.633
AMH (ng/mL)	8.77 ± 4.3	7.23 ± 3.4	.044	9.12 ± 4.34	6.82 ± 3.10	.011
PCOS phenotype			.069			.627
A	16 (45.7%)	172 (28.0%)		14 (48.3%)	45 (40.5%)	
B	0 (0.0%)	8 (1.3%)		0	1 (0.9%)	
D	19 (54.3%)	434 (70.7%)		15 (51.7%)	65 (58.6%)	
Controlled ovarian stimulation protocols			.229			.496
Follicular phase GnRH: a long-acting protocol	32 (91.4%)	569 (92.7%)		26 (89.7%)	101 (91.0%)	
Antagonist protocol	0 (0.0%)	19 (3.1%)		0 (0.0%)	4 (3.6%)	
Luteal phase GnRH: a short-acting protocol	2 (5.7%)	12 (2.0%)		2 (6.9%)	3 (2.7%)	
Stimulation protocol	1 (2.9%)	14 (2.3%)		1 (3.4%)	3 (2.7%)	
The number of embryos transferred			.177			.251
Single embryo	20 (57.1%)	279 (45.4%)		16 (55.2%)	48 (43.2%)	
Double embryo	15 (42.9%)	335 (54.6%)		13 (44.8%)	63 (56.8%)	
Mode of insemination			.066			.904
IVF	26 (74.3%)	526 (85.7%)		22 (75.9%)	83 (74.8%)	
ICSI	9 (25.7%)	88 (14.3%)		7 (24.1%)	28 (25.2%)	
The type of embryos transferred			.594			.566
Cleavage	18 (51.4%)	344 (56.0%)		15 (51.7%)	64 (57.7%)	
Blastocyst	17 (48.6%)	270 (44.0%)		14 (48.3%)	47 (42.3%)	
Delivery			.002			.014
Vaginal	2 (5.7%)	184 (30%)		2 (6.9%)	32 (28.8%)	
Cesarean	33 (94.3%)	430 (70%)		27 (93.1%)	79 (71.2%)	
Gestational age (wk)	36.97 ± 2.8	38.52 ± 1.6	.002	37.03 ± 2.84	38.53 ± 1.52	.010
Endometrial thickness at the day of embryo transfer (mm)	12.77 ± 2.5	12.28 ± 2.5	.262	12.59 ± 2.53	12.19 ± 2.64	.468
Serum estradiol levels on the day of HCG injection (ng/mL)	3085.17 ± 1747.4	3430.72 ± 1771.8	.006	3214.65 ± 1849.96	3273.83 ± 1660.64	.868
Total Gn dosage (IU)	2564.4 ± 1081.5	2121.52 ± 912.3	.256	2424.31 ± 1058.77	2343.93 ± 1088.22	.722
Total oocytes retrieved	17.03 ± 7.505	15.95 ± 5.81	.407	17.55 ± 7.98	16.27 ± 6.83	.387
SBP (mm Hg)	122.51 ± 15.84	114.81 ± 10.15	.007	118.21 ± 11.94	119.00 ± 8.65	.739
DBP (mm Hg)	80.457 ± 10.98	73.554 ± 7.64	.001	77.34 ± 8.24	77.19 ± 7.14	.919
TG (mmol/L)	1.91 ± 1.64	1.37 ± 0.84	.058	1.91 ± 1.79	1.53 ± 1.02	.281
TC (mmol/L)	4.48 ± 0.68	4.28 ± 0.77	.126	4.48 ± 0.59	4.32 ± 0.85	.256
LDL (mmol/L)	2.72 ± 0.61	2.57 ± 0.72	.231	2.69 ± 0.52	2.64 ± 0.79	.637
HDL (mmol/L)	1.31 ± 0.34	1.37 ± 0.31	.266	1.33 ± 0.35	1.31 ± 0.27	.729
FBG (mmol/L)	5.14 ± 0.65	4.90 ± 0.48	.042	5.04 ± 0.65	4.88 ± 0.46	.214

Data are presented as mean ± standard deviation or n (%). PCOS phenotype includes 4 subtypes, namely, A, B, C, and D, which present with HA + PCOM + menstrual abnormalities, HA + menstrual abnormalities, HA + PCOM, and PCOM + menstrual abnormalities, respectively.

Abbreviations: AMH, anti-Müllerian hormone; BMI, body mass index; DBP, diastolic blood pressure; FBG, fasting blood glucose; Gn, gonadotropin; HA, hyperandrogenism; HCG, human chorionic gonadotropin; HDL, high-density lipoprotein; HMG, human menopausal gonadotropin; ICSI, intracytoplasmic sperm injection; IVF, in vitro fertilization; LDL, low-density lipoprotein; PCOS, polycystic ovary syndrome; SBP, systolic blood pressure; T, testosterone; TC, total cholesterol; TG, triglyceride.

^*a*^P values were calculated by Student t-test or chi-square test.

### Relationship Between Serum AMH and Gestational Hypertension Before PSM

**Figure 1.**
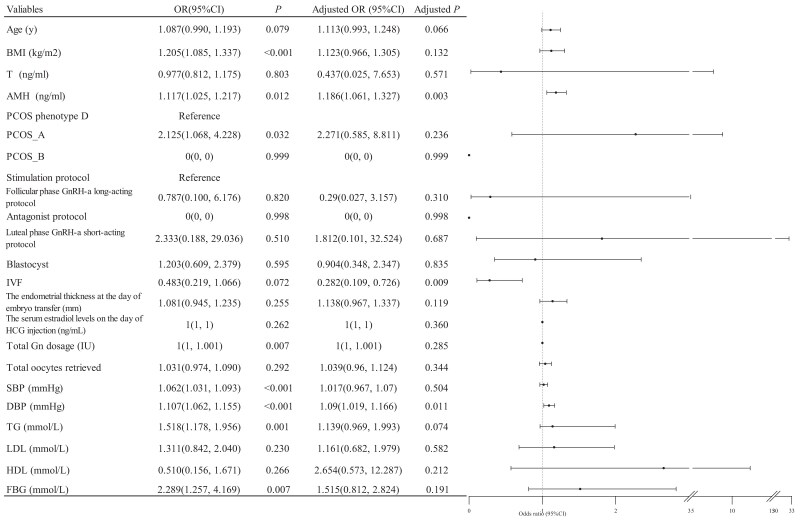
Key factors affecting the odds of gestational hypertension. Blastocyst is a stage in embryo development preimplantation that occurs around days 5 to 6 after insemination or ICSI. Adjusted for age, BMI, AMH, testosterone, controlled ovarian stimulation protocols, the type of embryo transferred, mode of insemination, the endometrial thickness on the day of embryo transfer, the serum estradiol levels on the day of HCG injection, total Gn dosage, total oocytes retrieved, PCOS phenotype, SBP, DBP, TG, LDL, HDL, and FBG.

In a single-factor logistic regression, the odds of gestational hypertension increased by 11.7% for every 1 ng/mL increase in AMH (odds ratio [OR], 1.117; 95% CI, 1.025-1.217; *P* = .012) (Fig. 1). Multivariable logistic regression was used to analyze the relationship between serum AMH and gestational hypertension. Multivariable logistic regression model included age, AMH, BMI, testosterone, controlled ovarian stimulation protocols, PCOS phenotype, the type of embryos transferred, mode of insemination, the endometrial thickness on the day of embryo transfer, the serum estradiol levels on the day of HCG injection, total Gn dosage, total oocytes retrieved, SBP, DBP, TG, LDL, HDL, and FBG. After adjusting for these variables, the odds of gestational hypertension was found to have increased to 18.6% for every 1ng/mL increase in AMH (adjusted OR, 1.186; 95% CI, 1.061-1.327; adjusted *P* = .003) (Fig. 1). IVF and DBP are also risk factors for gestational hypertension (Fig. 1).

The population was grouped by AMH percentile. Patients with PCOS and AMH levels above the 75th percentile (>9.30 ng/mL) were more common in the gestational hypertension group than in the without gestational hypertension group (OR, 2.1; 95% CI, 1.04-4.23; *P* = .035). Patients with PCOS and AMH levels above the 90th percentile (>12.31 ng/mL) were also more common in the gestational hypertension group than in the without gestational hypertension group (OR, 3.518; 95% CI, 1.57-4.89; *P* = .003). After adjusting for age, BMI, testosterone, controlled ovarian stimulation protocols, the type of embryo transferred, mode of insemination, the endometrial thickness on the day of embryo transfer, the serum estradiol levels on the day of HCG injection, total Gn dosage, total oocytes retrieved, PCOS phenotype, SBP, DBP, TG, HDL, LDL, and FBG, the odds of gestational hypertension increased by more than 2 times in the 75th percentile group (adjusted OR, 2.635; 95% CI, 1.132-6.137; adjusted *P* = .025) and by more than 3 times in the 90th percentile group (adjusted OR, 4.75; 95% CI, 1.672-13.495; adjusted *P* = .003) when compared to the without gestational hypertension group. There was no significant difference in patients with AMH levels above the 50th percentile (Table 2).

**Table 2.** AMH breakdown of PCOS patients with gestational hypertension and without gestational hypertension before propensity score matching

AMH (ng/mL)	With gestational hypertension n = 35	Without gestational hypertension n = 614	*P^a^*	OR (95% CI)	Adjusted *P^b^*	Adjusted OR (95% CI)*^b^*
Mean ± SD	8.77 ± 4.3	7.23 ± 3.4	.012	1.117 (1.025-1.217)	.003	1.186 (1.061-1.327)
>50th (>6.54 ng/mL)	20 (57.1%)	304 (49.5%)	.38	1.36 (0.683-2.705)	.168	1.823 (0.777-4.275)
>75th (>9.30 ng/mL)	14 (40%)	148 (24.1%)	.035	2.1 (1.04-4.23)	.025	2.635 (1.132-6.137)
>90th (>12.31 ng/mL)	9 (25.7%)	55 (9.0%)	.003	3.518 (1.57-4.89)	.003	4.75 (1.672-13.495)

Abbreviations: AMH, anti-Müllerian hormone; DBP, diastolic blood pressure; FBG, fasting blood glucose; Gn, gonadotropin; HCG, human chorionic gonadotropin; HDL, high-density lipoprotein; LDL, low-density lipoprotein; OR, odds ratio; PCOS, polycystic ovary syndrome; SBP, systolic blood pressure; TG, triglyceride.

^*a*^ *P* values were calculated by Student *t*-test or chi-square test.

^*b*^Adjusted for age, body mass index, testosterone, controlled ovarian stimulation protocols, the type of embryo transferred, mode of insemination, the endometrial thickness on the day of embryo transfer, the serum estradiol levels on the day of HCG injection, total Gn dosage, total oocytes retrieved, PCOS phenotype, SBP, DBP, TG, HDL, LDL, and FBG.

### Relationship Between Serum AMH and Gestational Hypertension After PSM

**Figure 2.**
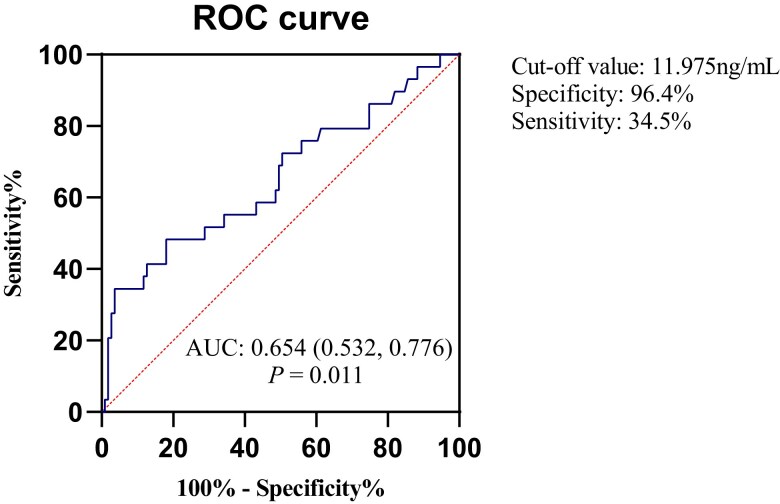
Receiver operating characteristic curves for AMH in the prediction of gestational hypertension after propensity score matching.

The baseline data of patients after PSM are shown in Table 1. After PSM, AMH level was still associated with gestational hypertension (OR, 1.186; 95% CI, 1.057-1.330; *P* = .004) (Table 3). ROC analysis evaluated the ability to predict gestational hypertension. The area under the curve (AUC) was 0.654 [95% CI (0.532, 0.776), *P* = .011] (Fig. 2). The optimal AMH cutoff value was 11.975 ng/mL with a specificity of 96.4% and sensitivity of 34.5%. We did an analysis of women without PCOS and added the results in Supplementary Tables S2-S4 (30). There was no significant relationship between serum AMH and gestational hypertension in patients without PCOS undergoing fresh embryo transfer.

**Table 3.** AMH breakdown of PCOS patients with gestational hypertension and without gestational hypertension after propensity score matching

AMH (ng/mL)	With gestational hypertension n = 29	Without gestational hypertensionn = 111	*P^a^*	OR (95% CI)*^a^*
Mean ± SD	9.12 ± 4.34	6.82 ± 3.10	.004	1.186 (1.057-1.330)
>50th (>6.80 ng/mL)	17 (64.3%)	53 (45.8%)	.297	1.550 (0.678-3.547)
>75th (>8.59 ng/mL)	14 (48.3%)	21 (18.9%)	.001	4.000 (1.677-9.543)
>90th (>12.06 ng/mL)	10 (34.5%)	4 (3.6%)	<.001	14.079 (4.001-49.536)

Abbreviations: AMH, anti-Müllerian hormone; OR, odds ratio; PCOS, polycystic ovary syndrome; SD, standard deviation.

^*a*^ *P* values were calculated by chi-square test.

### Subgroup Analysis

There were only 200 patients with available fasting insulin in this study. Compared to women without gestational hypertension, women with gestational hypertension had higher fasting insulin levels, but the difference had no statistical significance (*P* = .167) (Supplementary Table S5) (31). Patients were divided into hyperinsulinemia (>15 uIU/mL) and normoinsulinemia (≤15 uIU/mL). This analysis showed the effect of the serum AMH on gestational hypertensin was consistent in the fasting insulin subgroups (*P* for interaction is .191) (Supplementary Table S6) (31).

## Discussion

In this study, high AMH levels were found to be associated with the development of gestational hypertension in patients with PCOS after IVF/ICSI fresh ET, especially when AMH >9.30 ng/mL.

### Relationship Between AMH and Gestational Hypertension

In our study, prepregnancy serum AMH levels were higher in patients with PCOS and gestational hypertension than in those without gestational hypertension. Many retrospective case-control studies have reported higher levels of AMH in pregnancy serum and cord blood in preeclampsia than in normotensive women. Erfani et al (32) found that the serum AMH levels in patients with preeclampsia were higher than those in women with normal pregnancies, whereas the AUC for predicting PE was lower (AUC = 0.54; 95% CI, 0.45-0.63; *P* = .40). However, they did not collect detailed data on the time interval between AMH measurements and pregnancy or the onset of PE, which can influence the results due to the dynamical variation of AMH during pregnancy. Birdir et al (19) found that the preeclampsia group had a higher AMH value between 11 and 13 weeks of gestation, and DiPrisco et al (20) reported elevated levels of AMH in the cord blood of infants with mothers who had preeclampsia. However, these abnormal levels of serum or cord blood during pregnancy have limited reference value for predicting and clinically preventing hypertensive disorders during pregnancy. Moreover, none of these studies addressed the ovarian reserve status of the study population. Only 1 study focused on women with PCOS and reported that high AMH levels in the second trimester of PCOS were associated with a low risk of hypertensive disorders during pregnancy (33), which is contrary to our results. That study included 7 female patients with hypertension during pregnancy, with a prevalence of 4.4%, which is lower than the expected rate of gestational hypertension. In our study, the incidence of gestational hypertension in patients with PCOS was 5.39%, which is similar to that reported in previous studies (34, 35).

### AMH May Lead to Gestational Hypertension by Affecting the Placental Function

Our study reported an increased odds of gestational hypertension with AMH levels higher than the 75th percentile group (>9.30 ng/mL). In addition to gestational hypertension, AMH is associated with other types of placental dysfunction disorders. Several studies have reported an association between AMH and preterm birth. AMH that was not decreased during pregnancy presented a high risk of preterm birth (36). Kaing et al (37) and Hu et al (6) found that high AMH in patients with PCOS was associated with preterm birth in artificial insemination and IVF embryo transfer cycles, respectively. They found that the AMH threshold >9.75 ng/mL and >9.3 ng/mL, respectively, had a high risk of preterm birth.

To date, there are some indications that AMH may be directly involved in trophoblast invasion. The AMH is a member of the TGF-β superfamily. TGF-β1 inhibits trophoblast proliferation and invasion through the TGF-β/Smad signaling pathway (38). AMH also regulates Smad phosphorylation through its specialized type II receptor, shares some biological roles with other TGF-β family members, and may be involved in aberrant trophoblast invasion (39). The increased expression of AMH in the lesions of endometriotic patients indicates that AMH may have an impact on interactions between endometrial cells (including adhesion and invasion) through the AMH/AMH receptor pathway and/or with other TGF-β family members (40). High AMH levels can also affect aromatase Cyp19a1 activity in the placenta during pregnancy, which promotes the formation of a hyperandrogenic environment, reduces the synthesis of oestrogen and progesterone, and impairs placental function (10). These indicate that an AMH level higher than this threshold may directly or indirectly impair placental function, causing adverse pregnancy outcomes.

Our study suggested that IVF were protective factors for gestational hypertension. Mechanical disruption of the zona pellucida during ICSI sperm insertion, changes in embryonic expression of L-selectin, or cytokines at embryo implantation may lead to abnormal placentation with oxidative stress, failure or delay in cytotrophoblast differentiation, and reduced extravillous cytotrophoblast invasion and intravascular invasion, ultimately leading to fetal intrauterine growth restriction or maternal preeclampsia (41, 42). In view of these factors, we suggest that ICSI may increase the odds of gestational hypertension.

Our study found that high AMH was associated with the development of gestational hypertension, but ROC analysis showed that serum AMH level had limited predictive value for gestational hypertension (AUC = 0.654) and that the sensitivity of the optimal AMH cutoff value was only 34.5%. The possible reason is that PCOS phenotype and metabolic abnormalities, such as insulin resistance, obesity, and hyperandrogenism caused by PCOS itself, have affected the results. Therefore, in the future, serum AMH and PCOS phenotypes and metabolic risk indicators should be further combined to evaluate the risk of gestational hypertension in the PCOS population.

### Strengths and Limitations

Our study has several strengths. First, it is the first study to investigate the relationship between prepregnancy AMH levels and gestational hypertension in patients with PCOS and determine the odds of gestational hypertension in patients with high AMH levels. Second, AMH measurement of all patients was performed before the controlled ovarian stimulation in our center, making the results comparable between participants.

Nevertheless, this study had some limitations. First, it was a retrospective study with a small sample size. Second, patients with PCOS undergoing “all embryo freezing” because of ovarian hyperstimulation were not analyzed in this study; therefore, the results cannot be generalized to all patients with PCOS. Third, visceral obesity is a common feature in women with PCOS and is associated with increased risks of pregnancy-induced hypertension and preeclampsia (3), but visceral obesity was not assessed in this study. A larger study population and more detailed metabolic risk assessment (lipids, hemoglobin A1c, visceral obesity, etc.) are needed to further evaluate the relationship between AMH and gestational hypertension in the PCOS population. And compared with standard mercury sphygmomanometers, the electronic wrist blood pressure monitor may have introduced some bias in data obtained when the patients took health examinations during their first visits. However, the primary outcome in this study, gestational hypertension, was diagnosed by professional obstetricians using standard sphygmomanometry.

## Conclusions

The results of this study suggest that AMH levels are high in patients with PCOS and gestational hypertension after IVF/ICSI fresh ET. These patients with PCOS and high AMH levels before pregnancy are at high odds for gestational hypertension.

## Data Availability

The data that support the findings of this study are available upon request from the corresponding author. The data are not publicly available due to privacy or ethical restrictions.

## Abbreviations

AMH: anti-Müllerian hormone
ART: assisted reproductive technology
AUC: area under the curve
BMI: body mass index
DBP: diastolic blood pressure
ET: embryo transfer
FBG: fasting blood glucose
Gn: gonadotropin
HA: hyperandrogenism
HCG: human chorionic gonadotropin
HDL: high-density lipoprotein
HDP: hypertensive disorders of pregnancy
ICSI: intracytoplasmic sperm injection
IVF: in vitro fertilization
LDL: low-density lipoprotein
OR: odds ratio
PCOM: polycystic ovarian morphology
PCOS: polycystic ovary syndrome
PE: preeclampsia
ROC: receiver operating characteristic
PSM: propensity score matching
SBP: systolic blood pressure
TC: total cholesterol
TG: triglyceride
